# Supplementary material for: The Impact of the Implementation of Preventive Measures Due to COVID-19 on Work Design and Early Childhood Professionals’ Well-Being—A Qualitative Study
Source: Int J Environ Res Public Health. 2022 Feb 3;19(3):1739. doi: 10.3390/ijerph19031739 (PMC8834838; doi:10.3390/ijerph19031739)
Supplement: Supplementary file 1 [file ijerph-19-01739-s001.zip › ijerph-1489127-supplementary.pdf]

Table S1. Consolidated criteria for reporting qualitative studies (COREQ) checklist[i].

**No. Item**	**Guide questions/description**	**Explanation/Reported in chapter**
**Domain 1: Research team and reflexivity**		
Personal Characteristics		
1. Interviewer/facilitator	Which author/s conducted the interview or focus group?	S.G.
2. Credentials	What were the researcher's credentials? E.g. PhD, MD	Title page, M.Sc. Psychology
3. Occupation	What was their occupation at the time of the study?	Research associate
4. Gender	Was the researcher male or female?	female
5. Experience and training	What experience or training did the researcher have?	Educational background in psychology and occupational health research,<br>practical experience, advanced training
Relationship with participants		
6. Relationship established	Was a relationship established prior to study commencement?	None other than providing study informational material (see also 2.2. Sampling and Recruitment Strategy)
7. Participant knowledge of the interviewer	What did the participants know about the researcher? e.g. personal goals, reasons for doing the research	Participants were aware of S.G.'s work as a research associate focusing on child care proceedings during COVID-19 (see also 2.2. Sampling and Recruitment Strategy)
8. Interviewer characteristics	What characteristics were reported about the inter viewer/facilitator? e.g. Bias, assumptions, reasons and interests in the research topic	No other characteristics were reported about the interviewer.
**Domain 2: study design**		
Theoretical framework		
9. Methodological orientation and Theory	What methodological orientation was stated to underpin the study? e.g. grounded theory, discourse analysis, ethnography, phenomenology, content	Qualitative content analysis by Mayring (see also 2.4. Analysis)

	analysis	
Participant selection		
10. Sampling	How were participants selected? e.g. purposive, convenience, consecutive, snowball	Purposive sampling and snowball (see also 2.2. Sampling and Recruitment Strategy)
11. Method of approach	How were participants approached? e.g. face-to-face, telephone, mail, email	E-Mail, telephone (see also 2.2. Sampling and Recruitment Strategy)
12. Sample size	How many participants were in the study?	27 child care managers (see also 2.5. Study Participants)
13. Non-participation	How many people refused to participate or dropped out? Reasons?	None
Setting		
14. Setting of data collection	Where was the data collected? e.g. home, clinic, workplace	N/A, interviews were conducted via telephone (see also 2.3. Semi-structured Interviews)
15. Presence of non-participants	Was anyone else present besides the participants and researchers?	No (see also 2.3. Semi-structured Interviews)
16. Description of sample	What are the important characteristics of the sample? e.g. demographic data, date	27 child care managers (mean age = 48 years ± 10.76; range = 30-63 years; 93% female) (see also 2.5. Study Participants)
Data collection		
17. Interview guide	Were questions, prompts, guides provided by the authors? Was it pilot tested?	Description provided in 2.3. Semi-structured Interviews, interview guide provided on individual request
18. Repeat interviews	Were repeat interviews carried out? If yes, how many?	No
19. Audio/visual recording	Did the research use audio or visual recording to collect the data?	Audio recording (see also 2.4. Analysis)
20. Field notes	Were field notes made during and/or after the inter view or focus group?	During the interview, S.G. took field notes documenting contextual information (e.g., interruptions, pauses, tone of voice, laughing) which, of course, was only possible in a limited way by phone

21. Duration	What was the duration of the interviews or focus group?	average 36 minutes (min: 20 minutes; max: 50 minutes) (see also 2.3. Semi-structured Interviews)
22. Data saturation	Was data saturation discussed?	Yes (see also 2.4. Analysis)
23. Transcripts returned	Were transcripts returned to participants for comment and/or correction?	No
**Domain 3: analysis and findings**		
Data analysis		
24. Number of data coders	How many data coders coded the data?	Two, S.G. and H.T. with S.G. as lead researcher (see also 2.4. Analysis)
25. Description of the coding tree	Did authors provide a description of the coding tree?	see Figure 1 and 2.4. Analysis
26. Derivation of themes	Were themes identified in advance or derived from the data?	Deductive coding led by research questions (4 dimensions), others derived inductively (see also 2.4. Analysis)
27. Software	What software, if applicable, was used to manage the data?	MAXQDA2020 (see also 2.5. Analysis)
28. Participant checking	Did participants provide feedback on the findings?	No
Reporting		
29. Quotations presented	Were participant quotations presented to illustrate the themes/findings? Was each quotation identified? e.g. participant number	Yes, presented in Supplementary Materials including participant numbers (see Table S1. Quotes)
30. Data and findings consistent	Was there consistency between the data presented and the findings?	Yes (see also 4.1. Main Findings)
31. Clarity of major themes	Were major themes clearly presented in the findings?	Yes, with each research question having an individual paragraph (see also 4.1. Main Findings including 4.1.1., 4.1.2., 4.1.3., and 4.1.4.)

32. Clarity of minor themes	Is there a description of diverse cases or discussion of minor themes?	Yes, throughout the results (see also 3. Results, particularly category “mental underload”)

[1] Tong A, Sainsbury P, Craig J. Consolidated criteria for reporting qualitative research (COREQ): a 32-item checklist for interviews and focus groups. International Journal for Quality in Health Care. 2007;19(6):349-357.

Table S2. Quotes.

Main categories<br>*sub-categories*	Example Quotes
**(1) Implementation of Preventive Measures in Child Care and Associated Changes**	
**Reduction of the duration of child care per child**	We actively approached parents and contacted each family. We talked about open questions. Checked for needs. Asked ourselves: What do individual families need right now? And then, we managed to find acceptable solutions for everyone. (Int14)
**Introduction of fixed group settings**	
Conditions for group clustering	My facility wrote a letter to the parents, announcing there would be groups now and that they could come to me and talk about their wishes, who their child wanted to be with and in which group, what other preferences they had and so on. […] We then assembled the groups based on these criteria. And it turned out that we have a group of girls and one of boys. This is what parents wished for and we were so stressed, we were just checking who to put with whom, what they want, but there were too many criteria, we could not additionally observe that. (Int23)
Separation of group settings indoors	So, each group can use a certain area or a gym either in the morning hours or in the afternoon. And our group rooms are relatively large so that you could say: Well, even if I stay in the group for one day, we have quite some space here, the children don't step on each other's toes. (Int14)<br><br>The corridors were blocked with different materials, so that the children could not reach the other areas, and the entries were separated to ensure a relatively safe setting. (Int17)<br><br>Each of our groups has its own bathroom, so they don't, the group settings don't mix at all when going to the bathroom. We also have one nappy-changing room shared by two groups. But since we only have few children who need diapers, our colleagues can actually coordinate well. (Int5)
Separation of group settings outdoors	With barrier tape, we divided the outside area in three sections, so far as it made sense, and our staff had to swap because five groups could not be outside at the same time, they always needed to take turns. (Int9)
**Compliance with a fixed minimum staffing standard**	My colleagues and I think that the lack of flexibility is the greatest problem. The ratio of staff is actually based on the constant and permanent possibility of representation, so that people can get a day off, for their vacation or in case of illness or an appointment,

	for example. These things are not really possible at the moment. You notice that. Strain among colleagues has definitely increased. We cannot act as flexible as before. We must stay and work in our designated groups. (Int2)
**Implementation of drop-offs and pick-ups outdoors**<br><br>*Spatial distance during drop-offs and pick-ups*	 <br><br>Well, the one-way system is similar to the ones you might recognize from zoos or parks, which means you have certain designated ways. We have also illustrated this, so that you can see, we have an illiterate person here, as well, so that you can find the way to the different groups. Well, you have all group symbols there. There are waiting areas in front of the groups, which are marked, so that parents know where they have enough space and that there is sufficient distance between everyone. (Int18)
Staggered schedule during drop-offs and pick-ups	It is in fact the case that we initially have an extensive organizational effort, making sure that each parent gets a slot, knows when to bring their child, thus prevent waiting times at the door, guaranteeing that there is no crowd we are responsible for. This actually works really well. We tried it out in the first week, adjusted in the second week and it has been working ever since, we never have too many people at the door. (Int14)
Behavior of staff and parents in drop-off and pick-up situation	This is something we have to point out to the parents, as well. They need to wear face masks when bringing their children, they should not always take turns. No changes but one set person that should drop the children off. (Int10)<br><br>Each group's respective colleague then goes to the front door, receives the child at the door, they go to the washroom first, the child washes its hands, they go to the coat racks, the child undresses, puts its slippers on, and then they go to their group. (Int6)
**Management of sickness absence and testing for COVID-19**<br><br>*Symptomatic children*	 <br><br><br><br>We are in fact really careful, for that matter. Especially since we are not only responsible for one child. I am responsible for 64 children and 20 employees, which is why we are very sensitive if someone has disease symptoms. We really hope for the parents' understanding. We explain the situation, again. "We know it seems silly, but you have to consider that we do not only care for your child. You would want the same if another child came here with these symptoms." So far, it has worked well, more or less. They often lack understanding because the symptoms are not the usual

	ones for COVID-19, the child is just unwell, has a runny nose. Then they often reply: "Well, a runny nose is not said to be one of the symptoms. Yes, well, your child is still sick. It should therefore stay at home." But I discuss these things because I think that I have to be responsible here and I somehow need to justify our ways. (Int14)<br><br>So, the issue was in fact very fast moving. Sometimes, we immediately called the parents, when there was a runny nose or some sneezing and we had the children picked up. And then, they had to be symptom-free for 48 hours. Naturally, this was a great challenge for their parents, especially for those, most of the parents work, for those who have to go to work. And this does not lead to much understanding either. Thus, I sometimes explained that we cannot change it, the land sets the regulations, and we have to observe them. Then they changed the rule to 24 hours shortly thereafter.(Int18)
Symptomatic staff	Employees naturally get sick. But in addition, due to corona, as soon as they have a cold or a sore throat or whatever symptoms like a dry cough, I have to withdraw them. They have to check if they have COVID-19 or not. I tend to miss these colleagues more than if some colleagues were to come to work with a bit of a sore throat, irrespective of corona. Thus, we have experienced that sometimes more colleagues cannot be present for a longer time, until their test result is hopefully negative. (Int19)
Routine testing strategy	There once was a notification of a corona-hotline, if you were having trouble getting an appointment. I basically gave the information to all colleagues, but I don't think that anyone ever used it. I am afraid, and many others are probably also concerned, of spending two hours after closing time in some wait loop to get such a test done. I guess this is a bit frightening. On the other hand, I don't think this could be done during work time, it does not work in terms of care time. (Int26)
**Restrictions in play for children**<br><br>*Stricter regulations for personal toys*	 <br>But in fact only under difficult circumstances, because there are children who need to have their toy, well to feel safer, especially children who have just joined us or children who have been staying at home for three months during Covid. And this is not so easy to handle. Frankly, we have an arrangement with some children, they bring their toy in a bag, and we put it in a box near them, so that they know it is there, at least, even though they cannot just get it out. This is some sort of compromise for me to somehow allow bringing toys. (Int6)
Reduction of play opportunities	And we reduced the number of games in the groups, reduced the number of books, the number of children in the corners, on construction rugs for example. They must use the gym alone,

	somebody has to supervise them to check what they are playing, or yes. (Int7)
**Redesign of catering and food service**	
Preparation of meals	And the cook always prepared one cart for each group. They could then fetch it and eat in their group room. (Int23)
Serving and distribution of meals and beverages	One colleague stays and eats with the children, the other one distributes the food. Everyone used to take their own dish, but due to hygiene measures, we serve it now. They just come to the cart and our colleague serves them whatever food they want to have. But since there is an empty group, we can have some people eat there, we can split up. And the other group uses the children's kitchen or rather the staff room for the children. So, this way, we can arrange the dining places. (Int8)
**Extension of hygiene and cleaning practices**<br><br>*Compliance with a hygiene and cleaning conceptual framework*	 <br><br><br>Well, I think it is certainly a different waking process, right, for the children and for us, simply with regard to hygiene standards, I really have to deal with that, retrospectively we have always had high standards, but they have increased even more, now. (Int12)<br><br>Realizing hygiene measures surely is and will stay the biggest challenge, yet thank goodness, we had more time in the afternoon, we are quite well equipped, staff-wise. One of our colleagues was some sort of hygiene officer, she was always paying attention, and all colleagues were fully informed about our hygiene concept. So, in the afternoon from three p.m. on, one employee of each group has implemented this concept. (Int15)
Adaptions in hygiene practices	We always have to disinfect toilet lids, toilet seats, when they have used the bathroom. Handles need to be disinfected, light switches, contact surfaces where children were sitting, everything has to be disinfected again and again. (Int10)
Modifications for external cleaning services	There is an incredible amount of checking needed. Examine if the designated company properly cleaned everything. If not, we have to do it. (Int1)
**Focusing on occupational health and safety (OHS)**<br><br>*Personal protective behavior and personal protective equipment (PPE)*	 <br><br><br>We can't observe distance regulations with the children, we can't manage that. And we actually keep the proper distance with other adults, we use face masks when communicating. (Int10)

Individual risk assessment / Occupational-medical health examinations	All employees belonging to a risk group were encouraged or had to check in with their general practitioner and then with their occupational physician in order to be able to work again.<br>Both parties had to agree before they could come back. (Int14)
Workplace risk assessment	This risk assessment, we got it from our employer and went through it. Well, it was practically made for our sector, as well. Well yes, certainly, we try to observe it down to the last detail, but it's not always possible in everyday life. (Int15)
**(2) Perception and evaluation of preventive measures and associated changes**	
**Vagueness and ambiguity of measures**	The handout always said: We should do it like this or that, unless it's not possible. Every third sentence said something like that. You should observe distance regulations, unless it's not possible. You should not fill in for someone in other groups, unless you have to. And so on. These things made me think: That just can't be true. (Int22)<br><br>In the meantime, I was so busy reading, and sometimes you just have to question if what you are reading makes sense after all. There are three pages of rules telling you what to observe and eventually it says, analogously speaking: "And if you cannot manage this staff-wise, you can neglect it." Is that so? I don't know, it is well-known that only few child care center have enough staff. Thus, it is already questionable, anyway. (Int24)
**Reasonableness of recommended measures**	We do that, yes, I think we do, but let's say there are two children playing on the construction play carpet, they don't keep a distance of 1.5m. I can't have a group of 20 children sitting together, with a 1.5m distance between them, there would not be enough space. This is reality, the children, well, this is why they come to a kindergarten, to play with others, as well. We have no cuddle corners anymore, we removed them, the ball pool was removed, too. But really, pulling tables apart, having personalized chairs, I really wonder what this is about. We disinfect all that in between, anyway, right? But I really wonder what the point is. (Int3)
**Discrepancy between measures in public and in child care**	Certainly, parents are discussing, because they are living according to what I have just described. And they tell us, everything is open now. We can go to the playground. What are these core groups about? This is part of the discussion. I think we are aware that Covid is still there. But it is good that there is so much normality for one thing, that you can go to the outdoor pool, do sports, visit the playground, and then you question these core groups. Because in real life, they eat ice cream together, they go to the playground, to the pool, they meet and get together and so on. Children meet up, right? So, for example if Max from one group and Julius from the other meet and play in the afternoon. They are not in the same core group. (Int3)

	Yes, in a child care center, we have to follow some rules that the outside world ignores. And this doesn't fit at all. Do I know whether parents might be walking through the old town with their children at the weekend? No, I don't. And we keep the children at a distance. For me, this does not make any sense at all. It is not over, yet. And it will become worse, but the others, I don't know, I only know for myself, what I am doing, I can't provide a guarantee for them. (Int7)<br><br>It is so annoying, everyone is acting as if these hygiene measures represent effective protection, but this is bullshit. They don't protect us. Because as soon as there are 22 children from 22 different families with, let's say five other families in the afternoon, we mustn't mix at all, but we all know that they play together outside. (Int17)
**Perceived benefits due to the measures**	And well, it is possible, since there are small groups and the children do not stay that long, due to that you can obviously concentrate more intensively on the children. Right? And there are always children who, let me put it this way, who tend to go lost in an open concept, because they suddenly like disappear, right? Getting lost in the masses. And those children are blessed, of course. So, you really notice that they are glad not to be in an open concept, right now. (Int9)<br><br>So, we had completely new experiences. Initially, the pedagogues were really enjoying the time in the groups. I have to mention that, too. They said that this group thing was kind of nice, too, that it had some advantages. And the children are comfortable. I personally think that this group concept worked well during corona. Because these groups convey a feeling of security to the children. And corona deeply unsettled all families and the children. Which is why this group concept worked well for pedagogues and children. (Int23)<br><br>And some kids suddenly like dressing and undressing, because their parents don't come to the coat racks with them. So, there is no prolongation anymore, like oh no, I can't do that now. And their mother or father quickly ties their shoes. Or unties them. In that sense, children are more independent now, I would say. (Int16)
**(3) Impact on Work Characteristics**	
**Work organization and workload**<br><br>*Reopening preparations: Increased workload*	 <br><br>And obviously, this was a challenge in times of corona, that areas had to be modified, areas with few options, since we do not work with chairs and tables in most rooms, now. […] And the bistro also had to, well according to the number of children there are five

and expenditure of time	groups now, a dining area had to be turned into another group, and all other groups had to be capacitated so that nobody had to eat on the floor, right? And this was a challenge, that you had to modify areas with inappropriate material or few options, like you simply didn't have them and you somehow had to organize seats for the children on a group level. (Int17)<br><br>And then, thank goodness, due to a cleaning room that we had to convert, again […]. So overall my employees and I had quite some long evenings of puzzling and worrying about how to solve all issues. (Int12)
Daily working routine: Increased workload and expenditure of time	The complete disinfecting process is obviously very time-consuming. So, this means disinfecting all surfaces after any meal or whenever you notice someone sneezing on them. These things already take up quite some time and space. (Int18)<br><br>We have to do so much more besides child care and that is really extensive and problematic: The issue of disinfection, and how are we supposed to have enough time for educational documentary, conversations with parents, team meetings? This is the most exhausting part. (Int27)
Noisy work environment	And now, we only have these four groups, and 20, 23 children have to stay in one room with only one small side room, compressed so to say. All children and colleagues are exposed to increased noise pollution. (Int6)
Overtime	I know, you have to distinguish there, well I like involving the parents and taking my time, this will of course/I have constantly worked, as well, I have done extra hours at the time, I haven't spent a single day at home, because I simply acted as a contact person. (Int17)<br><br>But we have really struggled and worked hard. I have worked way more than usual, too. Many of us have. We have invested a lot of time and energy to offer the children what they in fact received then. (Int23)
Interruption of work and multitasking	I always say, I would love to give 22 children to such a minister, he should have a party, accompany everyone to the bathroom, clean the seats, write an educational documentary on top and plan a support offer that precisely fits this group's needs. (Int17)<br><br>The small things consume the most time. It always sounds like nothing and when you think about it, I have done that, I have to write that down and if I use the computer, I have to wipe it clean for the next colleague, and these are the small things. (Int21)<br><br>Yes, constantly being bothered, since you always have to pick up the child personally and drop it off. This is something that we don't

	know in that sense. Somehow, yes, but it obviously means higher effort. (Int16)
Higher physical demands	But carrying the things by ourselves, yes we were supposed to drag them ourselves. (Int3)
**Work content and work tasks regarding pedagogy**<br><br>*Settling in of children during COVID-19*	 <br><br><br>We handle the settling in completely different now, too. If parents do come and have to stay in a different room, it means greater challenges for us. I need to get used to this and I really miss the old times. (Int27)<br><br>The transfer of the three-year-olds to the next group. We used to make it soft. We have always accompanied them. This process is rougher now. It is, when they come back after the holidays, all of a sudden, they are not in their group anymore, they are in the new group without really having understood it, I think. (Int7)
Intensified hygiene education for children	We also drew posters with the children, well for the children. So that everything is visually illustrated from the beginning, because we also had to learn that in fact. And we hung them up everywhere, for the parents, the children, in every group, where they wash their hands, it is always illustrated again, right? Therefore, painted by themselves and somehow laminated and put on the wall. Yes, and singing happy birthday twice, the children knew that from home, too. You can find that everywhere and they have internalized it quite well, but some things go wrong nevertheless. (Int15)
Enhanced pedagogical attentiveness and sensitivity	Many children here are frightened, many children that have come back. For them, this virus is not graspable, it's not visible. And many children are scared, or rather don't know how to deal with it. For me, an even bigger threat is that, for one thing, there are children who deal with it, but there are also children who don't, but they might be scared and worried, too. Their parents also told them, it makes people sick, some die of it. And these things are so surreal for children. (Int6).
Difficult compatibility of early childhood pedagogy and infection prevention	Well, in some parts, it obviously works, at lunch, for example. These changes concern the conceptual frame and can be understood by the children. Yet, a certain balance might be missing as soon as it's about the children's needs that are directly affected, sadness, food or thirst. The same also applies to the education in hygiene. At the moment, it's very difficult, going to the washroom in general. We decided that it should stay the same for the small ones. We partly neglect self-efficacy and self-confidence then, which would normally be conceptually implemented. It is often a bit of either this or that. It does not work perfectly, symbiotically. (Int2)

	Our work was not child-focused anymore. Right? It was only about the different requirements regarding corona. However, it was neither child-focused nor development-oriented. (Int11)
**Social interaction and cooperation**	
Changed interaction with parents	It was exciting to see how communication works, namely with the parents, with employees, the ones here on-site and those at home. It was more time-consuming than before or different at least. We usually prefer face-to-face conversations in passing, and now we knew that all information had to reach parents in other ways. Yet not everyone replied to e-mails. For me as child care manager, staying in touch is what I perceived as a great challenge during this time. (Int27)<br><br>Some of our families also live in an accommodation for refugees. There simply is no mailbox nearby. You somehow need to work things out, first. (Int20)
Changed interaction within the team	Interaction between all groups has always been very important for us, this is absolutely missing, and it certainly has an effect on the whole team, because regular exchange is just missing. We try to have our weekly team meetings. If possible, we sit outside or in the great hall, so that we can keep the distance. But it is not quite the same. We only discuss necessities. (Int26)<br><br>We reduced team meetings. We hold them in a multipurpose room. We have only had one meeting so far. Due to my function as a child care manager, I am in contact with all settings. I have, I think I have worked way too much, I've had a lot of extra work. All issues are discussed separately within each group. Obviously, joint decisions are still made via WhatsApp. (Int10)
**(4) Effects on Early Childhood Professionals' Well-being**	
**Mental underload due to the absence of children**	Honestly, some of our employees were unchallenged at times due to the small number of children. This problem has indeed never occurred before. It sounds a bit silly, but in the end, it feels unsatisfying for everyone, when there is not enough work. We were actually able take care of things that had been neglected before. We conceptually developed some issues or dealt with certain pedagogical topics again, things that all colleagues could work out. But it was somehow challenging to undertake different tasks or deal with other issues far away from the usual daily routine. (Int26)
**Worry about children staying at home**	In some cases, we were also worried about the children, of course, because they had to stay home alone for such a long time. Especially when families are not connected to the youth welfare office. There, we had no legal means at all, at first. (Int5)

**Emotional exhaustion, psychological and physical strain due to extra work and new responsibilities**	But this is done at the cost of all employees who already feel exhausted, they get more and more and it's just not possible to deal with it. (Int17)<br><br>You feel incredibly tired and exhausted. We received such a great pile from the ministry, and then again, we belong to the Joint Welfare Association and, well, everything related to it. And we always had to check for details to see if they had been changed for us, again and again. (Int4)
**Anger due to the lack of reward and appreciation**	And right at the beginning, I was very annoyed after Mrs. Merkel's press conference, because child care center had not been mentioned at all. They were only talking about schools. We discussed it. It was expectable that we were not even taken seriously as an educational institution. I have to say I find this very, very irritating. (Int13)<br><br>Have you noticed the number of newspaper articles on complaining parents who had to take care of their children? And in contrast to that, have you ever seen only one article about day carers or early childhood professionals complaining because children could not keep the proper distance, even though they knew of it? (Int 22)
**Reduced psychological sense of community and identification at work**	Well, the fact that everyone, I mean all the organizational aspects, to rearrange some benches, I don't know, separating certain rooms, that is no big deal, but the human aspect of a child care center, feeling comfortable, talking to each other, having time. This is omitted. We take the children and only communicate the most important issues. There is no talking in passing, no: "You look really great today!". Or how about "You look so tired"? The interpersonal is lost. And this is the worst. (Int7)<br><br>Yes, this was very conflictual, and it really bothered the relation between kindergarten and parental home. I think it will take some time working on that and trying to fix it. That was indeed very, very conflictual. (Int25)<br><br>These things do not seem very dramatic at that moment, but they have an effect little by little. It's just a pity. It's no big deal, no disaster, but you don't feel like being a great institution anymore. Something gets lost. People are rather busy in their own groups. (Int27)
**Feeling of being left alone and loss of control**	Well, we do not feel on board at all, we certainly feel left alone. Plainly spoken, we think it' more about the votes than what actually happens in the houses. (Int1)
**Perceived risk and fear of infection**	And all this uncertainty, at the beginning nothing was certain, then they said that children were not contagious at all, then they said that children were highly contagious, and this is something you have to deal with on a daily basis, right? I mean, you don't have

	time to think about that at work, but at home you start wondering, I have a family, others have families, too. Some of my employees have elder relatives, ill relatives, of course they are worried that they might bring something home. (Int24)
**Fears and insecurity about the future**	More children could come at different hours. We are supposed to open the whole institution again. And I am really concerned that I might not be able to comply with care times. That colleagues are absent, because fall is just approaching. This normally happens at a child care center with many children, the risk of infection generally increases. It happens every autumn, every winter. And we really have to wait and see if we can keep the minimum standard then. (Int19)

---

[i] Tong A, Sainsbury P, Craig J. Consolidated criteria for reporting qualitative research (COREQ): a 32-item checklist for interviews and focus groups. International Journal for Quality in Health Care. 2007;19(6):349-357.
